# Evaluation of the accuracy, occlusal contact and clinical applications of zirconia crowns using artificial intelligence design versus human design

**DOI:** 10.1016/j.jds.2025.03.016

**Published:** 2025-03-27

**Authors:** Che-Ming Liu, Tsung-Yueh Lu, Ching-Shuen Wang, Sheng-Wei Feng, Yu-Chieh Lin, Sheng-Yang Lee, Wei-Chun Lin

**Affiliations:** a Department of Dentistry, Wan-Fang Hospital, Taipei Medical University, Taipei, Taiwan; b School of Dentistry, College of Oral Medicine, Taipei Medical University, Taipei, Taiwan; c Division of Prosthodontics, Department of Dentistry, Taipei Medical University Hospital, Taipei, Taiwan; d Digital Medicine Center, Translational Health Research Institute, Vilnius University, Vilnius, Lithuania; e Institute of Research, Development and Innovation, IMU University, Kuala Lumpur, Malaysia; f School of Dental Technology, College of Oral Medicine, Taipei Medical University, Taipei, Taiwan

**Keywords:** Artificial intelligence, Contact, Zirconia crown, Accuracy, Dental design

## Abstract

**Background/purpose:**

The uniqueness of human teeth necessitated that dental restorations be customized primarily through extensive manual labor. Therefore, this study explored the potential of AI designed dental restorations for clinical applications.

**Materials and methods:**

Digital artificial design and AI design crown restorations were replicated 10 times, for a total of 20 samples. The zirconia crown restoration was completed by strengthening and glazing according to standard clinical procedures. Samples were digitally archived using a dental scanner to assess reproducibility, precision, and occlusion. The human trial portion included natural tooth preparations by clinical standards. Three participants each designed two crowns, resulting in a total of six crowns. Dental x-rays were used for image evaluation.

**Results:**

The 3D accuracy showed that stereolithography (STL) and scan files of the AI design group were 3.4 and 6.6 times lower than the digital group, respectively (*P* < 0.05). The space of the occlusal surface of the AI-designed crown was 1.8-times higher than that of the digital design (*P* < 0.05). Intraoral optical images demonstrated that the AI designed crown closely resembled the human-designed counterpart in appearance. Comparison of color distribution showed more differences on the buccal and lingual sides between the two design patterns.

**Conclusion:**

Clinical images indicate that the shape, precision, and space of AI designed crowns are comparable to those of digitally designed crowns. Despite the spatial differences in contact between AI designed and digitally designed crowns, the in vivo and in vitro test results demonstrated favorable realism and contact quality.

## Introduction

In the past, dental restorations were exclusively manually designed and fabricated.^1^, ^2^, ^3^ However, with the advent of industrial digital technology, dental clinics are gradually undergoing a digital transformation.^4^^,^^5^ Those findings affirmed the progressive growth of digitalization in dentistry and its contributions to improved clinical treatments.^6^, ^7^, ^8^, ^9^ However, both digitalization and traditional manufacturing processes of restorations require human involvement in the design and fabrication. This requirement consumes a significant amount of manpower and time. Therefore, reducing costs and enhancing the efficiency of dental restoration production represent current primary clinical challenges.

At present, digitalization is gradually progressing towards the development of artificial intelligence (AI). AI algorithms rely on extensive databases of big data for their calculations.^10^, ^11^, ^12^ By leveraging machines and technology, AI can assist in completing human tasks.^13^^,^^14^ Machine learning and deep learning combine data with results, allowing AI to further interpret and explain graphics and language. Additionally, machine learning can be utilized for calculations in AI's deep neural networks.^12^^,^^15^ AI can automatically analyze data to make judgments and produce results.^16^ Therefore, the introduction of AI may improve dental restorations that currently require significant manpower.

Moreover, the manual segmentation step in computer-aided design/computer-aided manufacturing (CAD/CAM) is highly time-consuming and prone to low repeatability, often limited by human judgment errors, which can negatively impact outcomes of restorative treatments.^17^ Deep learning driven by AI can help minimize the occurrence of such errors to the greatest extent possible. It improves success rates and achieves more-precise treatment results. However, despite being developed over 70 years ago, AI still has limitations. The diversity and complexity of dental restorations pose challenges to the advancement of AI in dentistry. Reinhard Chun Wang Chau et al. reported a study on the accuracy and feasibility of AI designed single-molar prostheses. The study utilized AI algorithms to generate tooth morphology. The results demonstrated the feasibility of AI designed single-molar prostheses. With further training and optimization of the algorithm, the accuracy of biomimetic AI designed prostheses could be further improved.^18^ Hao Ding et al. investigated AI based computations of crown morphology and mechanical properties. Their study indicated that AI (3D-DCGAN) could be used to design personalized crowns with high precision, replicating the morphology and biomechanics of natural teeth.^19^ This previous study showed that artificial intelligence-designed dental crowns are more time-saving than digital and traditional processes and have good reproducibility.^20^ However, further research is still needed for practical clinical application.

Therefore, this study aimed to investigate the disparities between AI and manual digitization in the dental restoration manufacturing process. The objective was to compare the accuracy and contact of in vivo restorations created using artificial digital techniques and AI automation. The suitability of AI designed dental restorations placed in the oral cavity was evaluated through medical imaging. This study hypothesized that AI designed dental restorations would not exhibit significant differences compared to digitally designed (human designed) restorations.

## Materials and methods

**Figure 1:**
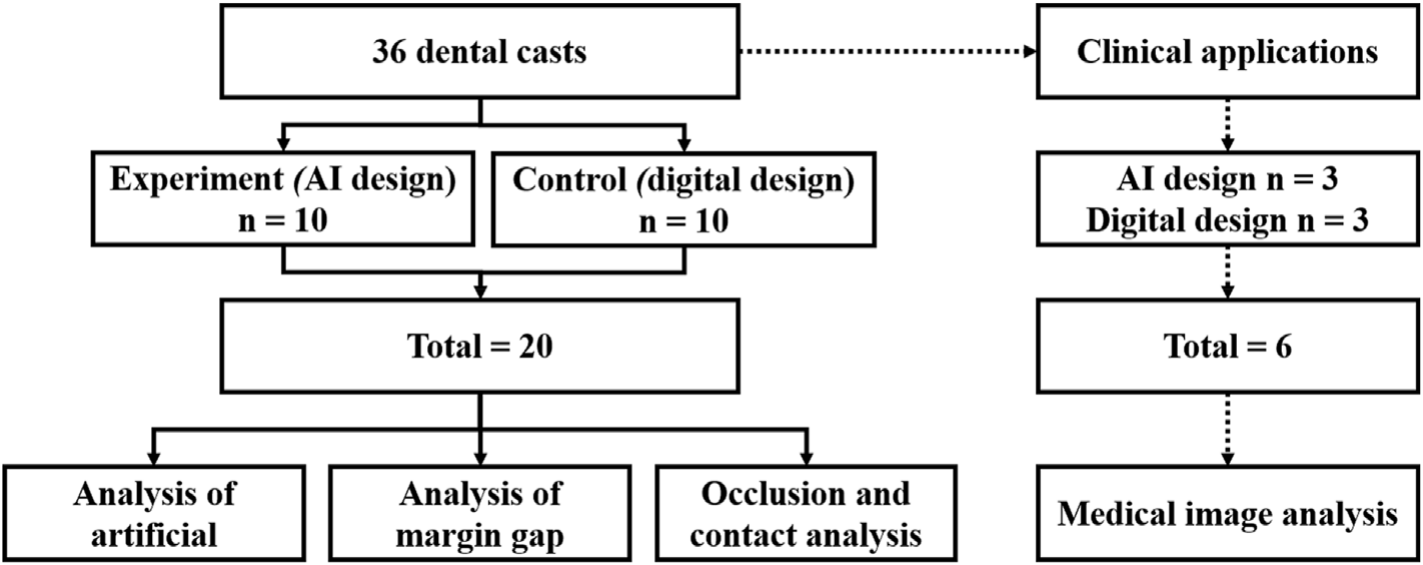
Experimental procedures and groups. The standard model comparison included files from a total of 20 M, consisting of 10 AI designed and 10 digitally designed crowns. The clinical application analysis compared files from a total of 6 M, comprising 3 AI designed and 3 digitally designed crowns.

The first molar on the left side of the mandible was selected as the model for a full crown restoration (Dental study model, Nissin Dental Product Inc., Kyoto, Japan). All groups were designed and manufactured using the same standard model and abutment teeth. This study was divided into two groups of design patterns (Fig. 1). A manual digital design was used to create a digital file of the model with a dental tabletop scanner (Medit T510, Medit, Seoul, Republic of Korea). Subsequently, the same dental technician used PrintIn DentDesign (Printin, Printin3D, Taoyuan, Taiwan) dental digital restoration software to repeat the restoration design 10 times. In the AI group, dental digital restoration software (Printin, Printin3D) was used for design based on previous research.^20^ The AI design in the PrintIn DentDesign software is computed using statistical models. The setting and modification including the tooth margin line were all performed by AI calculations.

All groups used zirconia (ST-Color, Upcera, Guangdong, China) to make crown restorations through CAM, and subsequent evaluations were conducted (N = 20). After the design of all groups was completed, digital files of the restorations were milled by Ceramill Motion 2 DNA (Amann Girrbach, Österreich, Koblach) and intensively sintered at 1530 °C for 8 h according to the manufacturer's instructions. This process resulted in the final zirconia crown restorations. An artificial digital design was used as a control group for analysis. After establishing digital archives of the restorations in this study, detection software (Medit Compare, Medit) was used to compare and analyze the surface accuracy.^3^^,^^21^ Finally, the compared images and data of different groups were recorded and analyzed as the results. The mean root mean squared (RMS) represented the overall surface difference of the sample.

**Figure 2:**
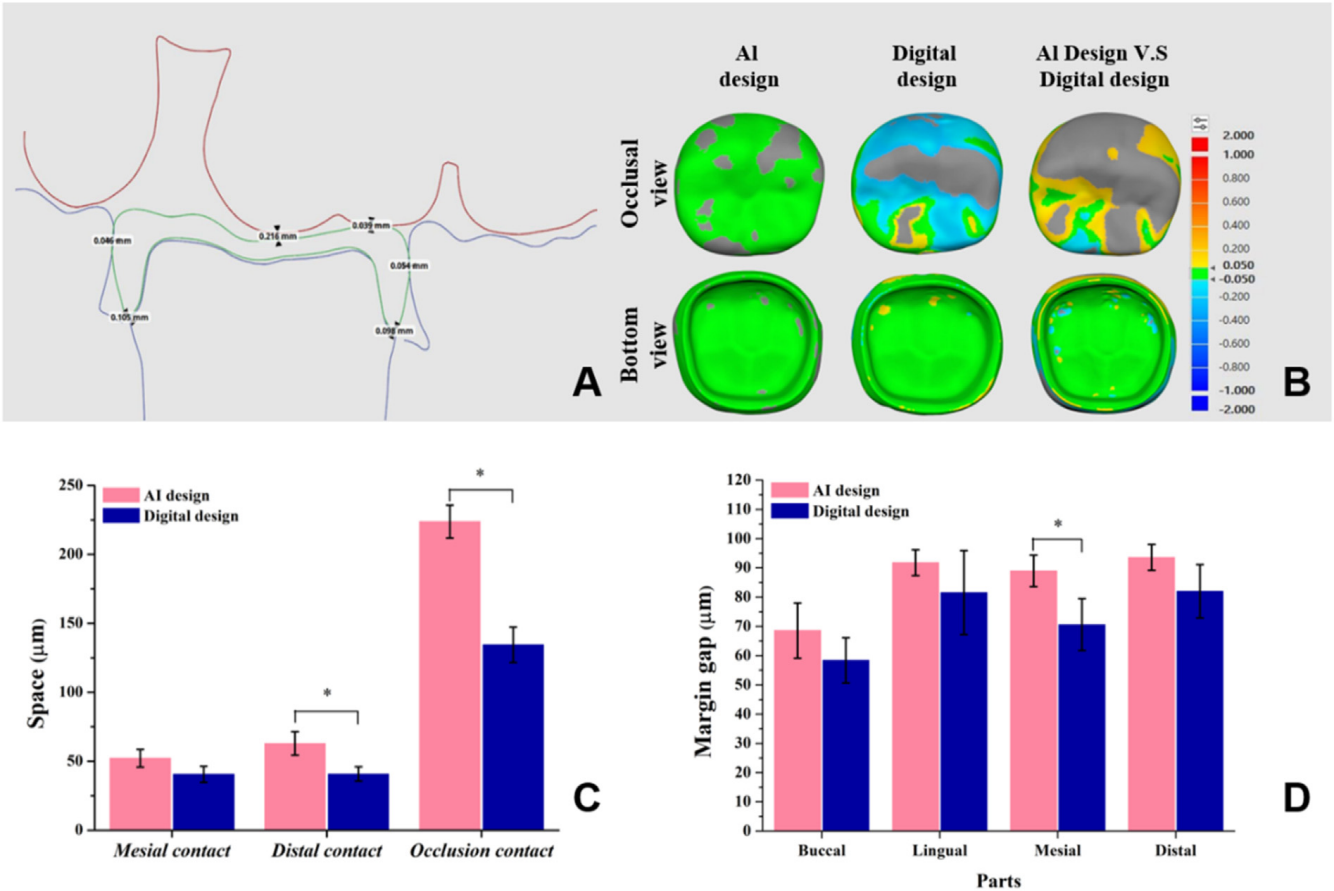
Surface reality and spatial analysis of dental casts. (A) Digital software analysis of the spatial schematic diagram of the crown. (B) Surface 3D trueness color distribution for different crown groups compared to each other. (C) Contact analysis of dental crown restorations. Means with ∗ were significantly different (*P* < 0.05, mean ± SD, n = 10). (D) Margin gap analysis of crown restorations at different positions. Means with ∗ were significantly different (*P* < 0.05, mean ± SD, n = 10).

The main model, the zirconia crown restoration, and the counter teeth were separately scanned using a desktop scanning machine (Medit T510, Medit). Then, detection software (Medit Compare, Medit) was used to import the upper and lower jaw models, abutment teeth, and full crowns. Finally, the software was used to slice sagittal sections and measure the margin gap, the distance between the prosthesis and adjacent teeth, and the occlusal height (Fig. 2A).

The clinical applicability of AI designed crowns was evaluated through human trials. Ethical approval was obtained from the Taipei Medical University Joint Institutional Review Board, Taiwan (TMU-JIRB No: N202109061). The primary inclusion criteria targeted individuals aged 20 to 70 who required molar crown restorations due to dental caries. The exclusion criteria included the following: (1) abutment teeth that had undergone root canal treatment or exhibited severe structural damage, (2) abutment teeth that lacked adjacent natural teeth on both sides, and (3) participants who had no opposing dentition. Ultimately, three participants were enrolled in this study for the preliminary evaluation of clinical applicability. To ensure uniformity in tooth preparation, all abutment teeth were trimmed with an average reduction of 1.5 mm, following standard clinical protocols. Each participant underwent both AI-designed and digitally designed restorations, adhering to the standard model protocol. In accordance with the fabrication process for clinical zirconia restorations, CAM milling, enhanced sintering, staining, and glazing were performed to complete the final restoration. During the fabrication of AI designed crowns, dental technicians did not intervene in the adjustment of marginal gaps, occlusion, or contact parameters. Their role was limited to removing zirconia supports and performing surface staining and glazing. Conversely, in the digital design workflow, dental technicians adjusted the marginal gaps, occlusion, and contact parameters before proceeding with support removal, surface staining, and glazing, following the same protocol as the AI designed crowns. The final restorations were analyzed for 3D accuracy, marginal gaps, occlusion, and contact.

This study used a hand-held optical camera (Galaxy Note20, Samsung, Suwon, South Korea) to observe and record differences in the topography of crown restorations loaded in the oral cavity. An image of the restoration cemented on a tooth was taken by dental x-ray for accuracy comparisons. The oral condition was photographed using a Bel-Ray II x-ray system (Belmont, Osaka, Japan) operating at 70 kVp and 10 mAs with a field-of-view to acquire planar x-ray images.

Data are presented as the mean ± standard deviation (SD) of 10 replicate samples. The clinical application analysis was conducted using data obtained from three patients. Data were analyzed using JMP 16 software (Statistics Analysis System, Cary, NC, USA). A two-way analysis of variance (ANOVA) with Tukey's honest significant difference (HSD) post-hoc test was employed to determine significant differences between groups, and statistical significance was set to *P* < 0.05.

## Results

Results of authenticity comparisons of 10 crowns made in all groups were 13∼120 μm (Table 1). RMS results showed that the stereolithography (STL) and scan files of the AI design group were 3.46- and 66-times lower than those of the digital group, respectively (*P* < 0.05). Results of comparing the two designs showed that digital and scanned files were 30–71 μm, respectively. The color distribution of the 3D accuracy of the crown showed that the AI design presented a green color with little difference on the surface and inside (Fig. 2B).

**Table 1:** Comparison of the surface accuracy of AI design and digital design dental crown restorations.

Types	STL file (*μ*m)	Scan file (*μ*m)	*P value∗*
	Mean RMS (SD)	Mean RMS (SD)	
Al design	13 (±3)^E^	18 (±2)^E^	.2712
Digital design	45 (±3)^C^	120 (±15)^A^	.0001***∗***
Al design V.S Digital design	30 (±4)^D^	71 (±7)^B^	.0001***∗***
*P value∗*	.0047***∗***	.0001***∗***	

STL: Stereolithography.

RMS: Root mean squared.

SD: Standard deviation.

AI design referred to the automated generation of crown morphology and the completion of the design process through an artificial intelligence system. Digital design referred to the process in which dental technicians used digital software to generate crown morphology and complete the design.

*P* value∗ <0.05 represents a significant difference.

Different capital letters indicate differences between groups.

The edge contact range between the proximal and distal surfaces of the two designs of crowns was 48∼52 μm (Fig. 2C). The distal surface contact of AI-designed crowns was significantly greater than that of digitally-designed crowns (*P* < 0.05). The occlusal distance results showed that the difference between the AI-designed and digitally-designed crown was 1.8-times (*P* < 0.05). Margin gaps of AI-designed and digitally designed crowns ranged from 57 to 92 μm in different tooth orientations (Fig. 2D).

**Figure 3:**
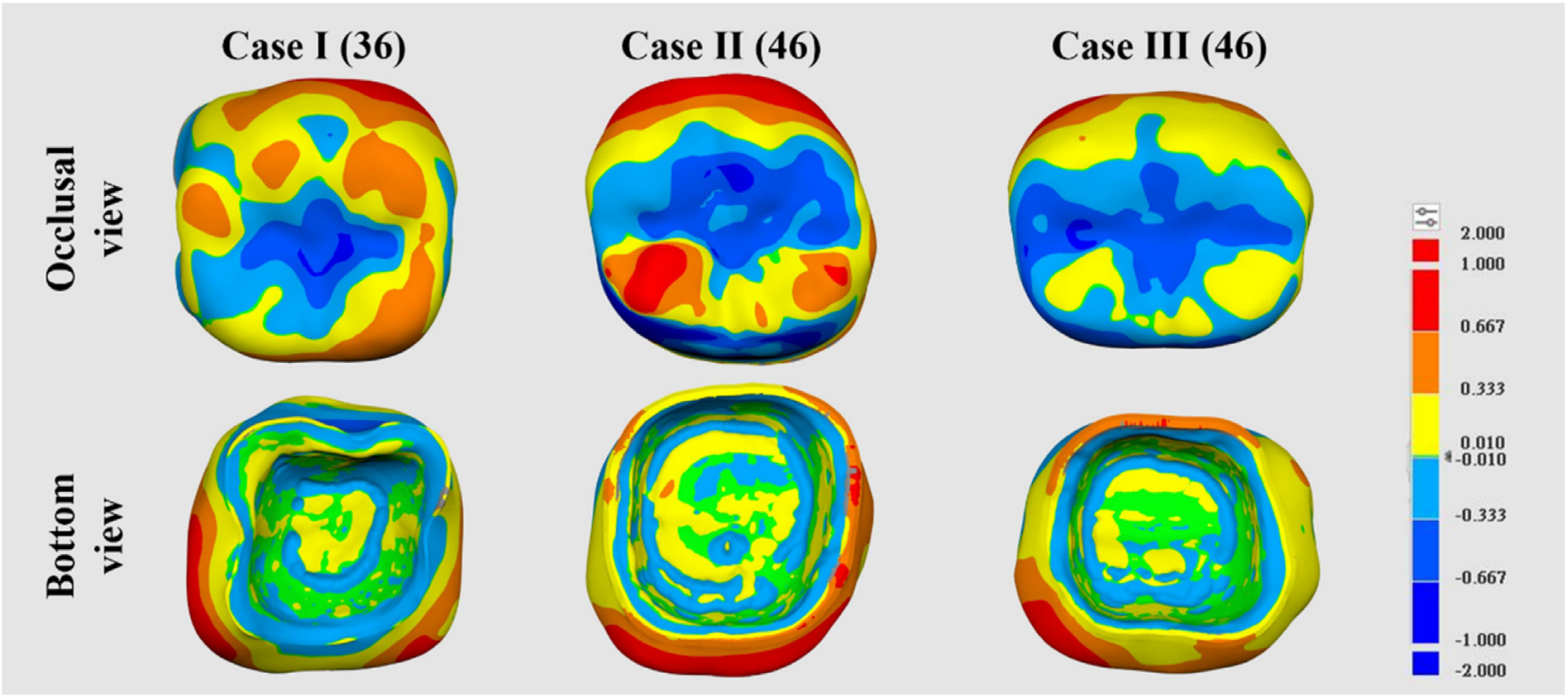
Representative color maps illustrated the differences in crown morphology between the AI designed and digitally designed crowns.

In this study, three clinical subjects who underwent dental caries treatment on the lower first molar were selected for in vivo trials. A notable difference between the two design patterns was observed in the color distribution of the crowns on the buccal and lingual surfaces (Fig. 3). Table 2 presents the measurement of RMS values for the three clinical cases, which ranged 189∼252 μm (*P* < 0.05). The mesial contact gap ranged −30 to 115 μm for AI designed crowns, while the digitally designed crowns ranged −79 to 4 μm (Table 3). Contact gaps on the distal surface between AI designed crowns and digitally designed crowns were found to be −56 to 16 and -53 to 23 μm, respectively (*P* < 0.05). The occlusal space of the AI designed crown ranged −356 to −141 μm, while the digitally designed crown ranged −184 to 134 μm. Overall margin gaps of the three groups of AI designed crowns were measured at 332.0 (±32) μm to 376.0 (±37) μm. On the other hand, overall marginal gaps of digitally designed crowns were 276.0 (±25) μm to 341.0 (±24) μm.

**Table 2:** Comparison of surface accuracy values between AI designed and digitally designed crowns.

Group	Mean RMS	Standard deviation
Case I	202^B^	10
Case II	252^A^	13
Case III	189^B^	11
*P value∗*	.0001***∗***	

RMS: Root mean squared.

*P* value∗ <0.05 represents a significant difference.

Different capital letters indicate differences between groups.

**Table 3:** Comparison of mesial contact, distal contact, occlusion contact and margin gap between AI designed and digitally designed crowns.

Position	Group	Al design Mean (SD)	Digital design Mean (SD)	*P value∗*
Mesial contact (μm)	Case I	94.7 (±7.4)^B^	−55.3 (±2.5)^E^	.0001***∗***
	Case II	115.0 (±7.0)^A^	4.7 (±1.5)^C^	
	Case III	−30.0 (±6.1)^D^	−79.3 (±12.9)^F^	
Distal contact (μm)	Case I	16.3 (±2.1)^B^	−53.7 (±9.1)^C^	.0001***∗***
	Case II	−56.0 (±6.0)^C^	23.7 (±3.2)^B^	
	Case III	37.3 (±5.5)^A^	15.7 (±3.1)^B^	
Occlusion contact (μm)	Case I	−283.0 (±36.0)^D^	−184.7 (±26.5)^C^	.0001***∗***
	Case II	−141.3 (±37.4)^C^	134.7 (±16.8)^A^	
	Case III	−356.7 (±38.2)^D^	−25.7 (±5.1)^B^	
Margin gap (μm)	Case I	332.0 (±32)^C^	276.0 (±25)^D^	.0001***∗***
	Case II	376.0 (±37)^A^	341.0 (±24)^C^	
	Case III	366.0 (±16)^B^	293.0 (±18)^D^	

Each contact space was calculated by measuring three locations, and the mean and standard deviation were determined accordingly.

SD: Standard deviation.

*P* value∗ <0.05 represents a significant difference.

Different capital letters indicate differences between groups.

**Figure 4:**
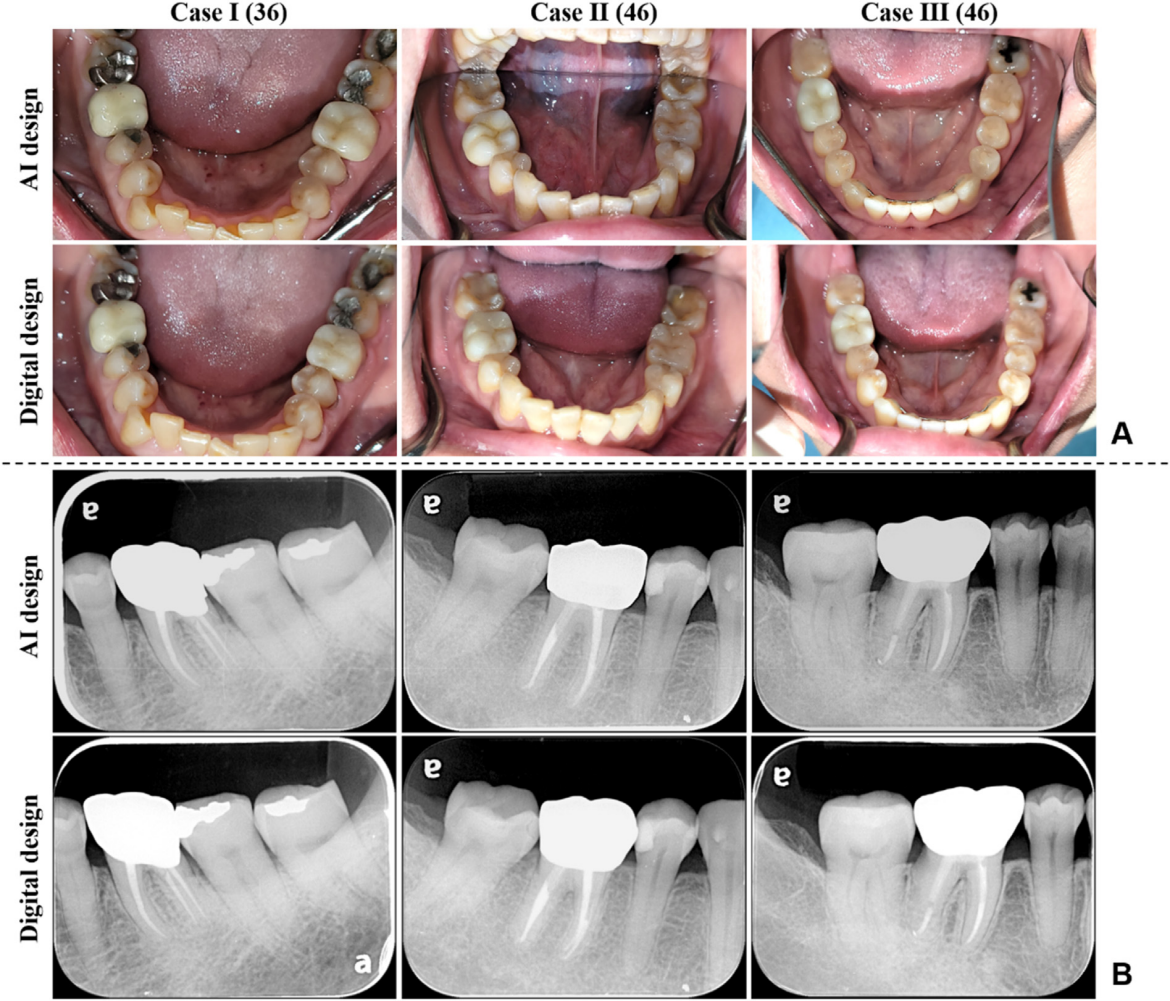
Medical image analysis of prosthetics installed in the oral cavity of subjects in human trials. (A) The optical image of the crown in the mouth is recorded by the camera. (B) Through the dental X-ray record the penetrating images of the crown in the mouth.

Fig. 4A presents an oral optical photograph showcasing three groups of clinical crowns. The contact between the crown and tooth edge of the abutment appeared to be a snug fit based on dental x-ray photography (Fig. 4B).

## Discussion

The unique structure of the human oral cavity contributes to the complex design of dental restorations, resulting in low accuracy and reproducibility.^22^ Therefore, clinical dental restorations often rely on manually customized design and fabrication. 3D accuracy mainly evaluates surface differences and reproducibility of crowns. In the AI design group, the RMS value for the intra-group comparison of the 10 crowns was the lowest, indicating high accuracy and similarity in each crown's design. In contrast, intra-group comparisons of the digital design group showed higher variations, suggesting that even if the same dental technician designs the same tooth, differences can occur. When comparing AI designs with digital designs, RMS values obtained from the STL or scan files fell within a single group (Table 1). It was observed from the color distribution that the interior of the crown exhibited good accuracy, while the main differences lay in the surface of the crown (Fig. 2B). Results indicated that the AI-designed crown closely approximated the 3D accuracy and shape of digitally designed crowns.

Adjacent areas, occlusal height, and the shape of the dental restoration directly impact the comfort of patients.^23^ Dentists clinically employ dental floss as a method to measure contact points of restorations. Ideally, dental floss should easily fit into the spaces.^24^ Dental technicians utilize occlusal contact registration strips to assess crown abutment and occlusal contact. When 40-μm occlusal paper is inserted between the crown, adjacent teeth, and opposing teeth, it should smoothly slide out, indicating an ideal scenario. Additionally, previous studies indicated that the occlusal clearance of crown restorations ranged approximately 460–520 μm.^25^ In this study, AI designed crowns appeared to have greater clearance compared to digital ones (Fig. 2C). This could be attributed to the AI algorithm considering the highest (the most prominent) point during the calculation process, resulting in excess space. Nonetheless, the designed crowns in both groups fell within an acceptable range. The previous literature discussed AI calculations of natural tooth appearance,^26^ with limited exploration in the context of restoration design and contact relationships. However, the contact relationship of a crown in restorative dentistry significantly impacts its success on natural teeth and its functional performance.

Teeth affected by caries often lose their protective enamel, increasing the risk of bacterial erosion and issues of subsequent secondary caries.^27^^,^^28^ Currently, the clinical requirement for the margin gap of dental restorations is < 120 μm.^29^ Both the AI designed and digitally designed crowns in this study exhibited similar margin gaps that met clinical standards (<120 μm) (Fig. 2D). Notably, the AI designed crown mainly differed in its appearance compared to the digitally designed crown (Fig. 2B). As a result, it demonstrated favorable adhesion performance at the margins.

Previous studies primarily relied on dental models for evaluating dental restorations.^30^ The surface analysis of clinical crowns in this study yielded results similar to those obtained from the dental models. However, there were differences between AI designed and digitally designed crowns (Fig. 3). The natural abutment tooth surface was uneven and lacked the smoothness of the dental models. Consequently, the distribution of errors on the mesial side of the crown was more prominent compared to the standard model. These findings indirectly impacted the overall RMS value (Table 2). Moreover, evaluating the marginal contact relationship between the restoration and the natural teeth is clinically important. A negative value indicates excessive contact, which can impede the proper loading of the crown. Conversely, insufficient contact can result in food impaction and excessive irritation of the gums.^24^ Clinical findings revealed that the digitally designed crowns exhibited a smaller space, necessitating more time for intraoral adjustment (Table 3). While the AI-designed crown displayed a larger occlusal space on the model, the opposite was observed in the oral cavity (Table 3). Natural teeth undergo positional changes and alignment over time.^31^ Hence, assessing a crown's performance in a patient's mouth provides a better understanding of its applicability. The AI crown design involved automatically capturing the boundary of the abutment tooth through software to establish the margin line. However, when a dentist trims a tooth, the edge line might not be a smooth, straight line. Consequently, during automatic measurements by software, the point-to-point connection may result in height differences, leading to an uneven edge line. Crowns from both design groups met clinical requirements, measuring <90 μm in all four dimensions (Table 3). This implies that both the AI automatic measurement of the margin line and the digital design achieved a precise fit.

The goal of clinical dental restorations is to restore oral aesthetics and function.^32^ Teeth vary in shape, size, arrangement, and color.^33^ The appearance is the most direct indication of the suitability of a restoration. The appearance of the AI designed crown was similar to that of the digital design (Fig. 4A). Its size and morphology did not differ from the optically illuminated surface in the mouth. A penetrating image analysis can clearly observe the accuracy of restorations and tooth margins without destroying samples. The contact relationship and margin fit of the crown observed through dental x-rays were consistent with the measured values. No excessive gaps were observed on penetrating radiographs of either crown (Fig. 4B). The past literature showed that when the marginal gap is too large, bacteria will remain at the junction and cause secondary caries.^28^ This shows that the AI designed crown had good appearance and margin fit.

The fabrication of dental crowns, which included both design and manufacturing, was a key component of dental technician education.^34^ As the precision of digital manufacturing equipment had reached a highly advanced level, the primary challenge lay in crown design. The design of a crown directly influenced the subsequent intraoral procedures performed by dentists, including crown fit and necessary adjustments. In fact, these factors not only affected clinical chair time but also impacted the confidence levels of both patients and practitioners. Therefore, regardless of whether crowns were designed by AI or human digital designers, the ultimate goal remained the creation of crowns that required minimal or no adjustment.^35^^,^^36^

However, crown design was inherently part of the overall treatment plan. With the gradual increase in dental artificial intelligence software.^37^ The integration of AI in this process raised ethical and medical considerations that required careful attention. Previous research indicated that AI applications in dentistry ultimately needed to be supervised by dentists, who served as the final gatekeepers to ensure patient rights and clinical efficacy.^38^ Thus, while AI generated crowns could be optimized to match a patient's oral conditions as accurately as possible, they still required dental professionals to oversee the final outcome and make necessary refinements.

Based on the findings, AI designed crowns demonstrated good accuracy and fit. The clinical applicability assessment revealed that, aside from differences in crown morphology, spatial design discrepancies existed between AI designed and digitally designed crowns. Therefore, the study's hypothesis was rejected. Nevertheless, both AI-designed and digitally designed crowns met the clinical requirements for crown restorations, indicating that AI designed crowns had significant potential for clinical application. A limitation of this study was that the sample size for clinical applicability was not sufficient to fully establish the feasibility of AI designed crowns for widespread clinical use. Therefore, further clinical validation was necessary to better reflect real clinical conditions.

## Declaration of competing interest

The authors have no conflicts of interest relevant to this article.
